# Programmed Cell Death Protein 1 Blockade Reduces Glycogen Synthase Kinase 3β Activity and Tau Hyperphosphorylation in Alzheimer’s Disease Mouse Models

**DOI:** 10.3389/fcell.2021.769229

**Published:** 2021-12-16

**Authors:** Yulian Zou, Chen-Ling Gan, Zhiming Xin, Hai-Tao Zhang, Qi Zhang, Tae Ho Lee, Xiaodong Pan, Zhou Chen

**Affiliations:** ^1^ Institute of Immunotherapy, Fujian Medical University, Fuzhou, China; ^2^ Fujian Key Laboratory of Translational Research in Cancer and Neurodegenerative Diseases, Institute for Translational Medicine, School of Basic Medical Sciences, Fujian Medical University, Fuzhou, China; ^3^ School of Pharmacy, Fujian Medical University, Fuzhou, China; ^4^ Fujian Center for Safety Evaluation of New Drug, Fujian Medical University, Fuzhou, China; ^5^ Key Laboratory of Technical Evaluation of Fertility Regulation for Non-Human Primate, National Health Commission, Fujian Maternity and Child Health Hospital, Affiliated Hospital of Fujian Medical University, Fuzhou, China; ^6^ Department of Neurology, Fujian Institute of Geriatrics, Fujian Medical University Union Hospital, Fuzhou, China

**Keywords:** PD1, PDL1, GSK3β, Aβ, tau hyperphosphorylation, APP/PS1, 5×FAD

## Abstract

Alzheimer’s disease (AD) is a central nervous system degenerative disease, with no effective treatment to date. Administration of immune checkpoint inhibitors significantly reduces neuronal damage and tau hyperphosphorylation in AD, but the specific mechanism is unclear. Here, we found that programmed cell death-receptor 1 (PD1) and its ligand PDL1 were induced by an intracerebroventricular injection of amyloid-β; they were significantly upregulated in the brains of APP/PS1, 5×FAD mice and in SH-SY5Y-APP cell line compared with control. The PD1 and PDL1 levels positively correlated with the glycogen synthase kinase 3 beta (GSK3β) activity in various AD mouse models, and the PDL1-GSK3β immune complex was found in the brain. The application of PD1-blocking antibody reduced tau hyperphosphorylation and GSK3β activity and prevented memory impairments. Mechanistically, we identified PD1 as a critical regulator of GSK3β activity. These results suggest that the immune regulation of the PD1/PDL1 axis is closely involved in AD.

## Introduction

According to an epidemiological report, approximately 50 million elderly people suffer from different degrees of dementia, and this figure is likely to rise to 150 million by 2050 (Peprah and McCormack, 2019). As one of the main forms of dementia, Alzheimer’s disease (AD) is a degenerative disease of the central nervous system characterized by progressive cognitive impairment and memory deficit, which poses a major public health threat worldwide (Berger et al., 2020). According to the amyloid hypothesis (Haass and Selkoe, 1993; Selkoe and Hardy, 2016), accumulated extracellular amyloid-β (Aβ) peptide is the primary contributor to the disease and one of the hallmarks of AD (Bloom, 2014). Glycogen synthase kinase 3 beta (GSK3β) is overactivated in AD (such as Aβ insult), thereby contributing to its progression (Takashima et al., 1996a; Takashima et al., 1996b); it is considered a marker for neurodegeneration in AD (Takashima, 2006). In the brain, GSK3β activity is associated with the generation of several phosphorylation sites on tau, as identified in *in vitro* and *in vivo* studies on AD (Leroy et al., 2007). More than 36 residues in tau are phosphorylated by GSK3β (Hanger et al., 2007), with Thr231 and Ser396 being the major phosphorylation sites (Billingsley and Kincaid, 1997; Li et al., 2006; Li and Paudel, 2006; Leroy et al., 2010; Moszczynski et al., 2015).

Programmed cell death protein 1 (PD1) is an inhibitory receptor on antigen-activated T cells. PD1, together with its ligand PDL1, constitutes a critical component in the induction and maintenance of autoimmune tolerance (Fife and Pauken, 2011). The PD1/PDL1 axis is an extensively studied immune checkpoint worldwide. Inhibition of the interaction between PD1 and PDL1 enhances T-cell response and confers potent anti-tumor activity (Brahmer et al., 2012). Increased interaction of inactivated GSK3β with PDL1 has been found in basal-like breast cancer (Li et al., 2016), and GSK3α/β inactivation blocks PD1 expression in CD8^+^ cytotoxic T lymphocytes (CTLs) and enhances immunity through PD1 downregulation (Taylor et al., 2016). These results suggest a close relationship between the PD1/PDL1 axis and GSK3β activity (Schulz et al., 2019). Despite extensive research on PD1 in tumor and immune cells, the mechanism of PD1 signaling in the central nervous system is largely unclear. PD1 is considered an inducible protein, and its expression is mainly limited to the thymus (Ishida et al., 1992). Functional PD1 has also been found in the dorsal root ganglion (Ishida et al., 1992) and other brain regions such as the thalamic and cortical neurons (Jiang et al., 2020), suggesting a neuronal role in the brain. Although PD1/PDL1 blockade reportedly exerts strong anti-AD effects, the expression of PD1 and PDL1 in the brain of AD mouse model is still unclear. However, a reduction in tau hyperphosphorylation has been reported in an AD mouse model after blocking antibodies of the PD1/PDL1 axis (Baruch et al., 2016; Rosenzweig et al., 2019; Schwartz et al., 2019), suggesting that PD1 blockade may regulate tau hyperphosphorylation by affecting the activity of some key kinases. Even though the PDL1/GSK3β immune complex has been observed in tumor cells, it is still unclear whether this interaction occurs in the brain and whether GSK3β is the direct downstream target of PD1/PDL1 to regulate tau hyperphosphorylation in AD.

In the present study, we demonstrated that PD1 and PDL1 are upregulated in AD models *in vitro* and *in vivo*. We also showed that the PDL1/GSK3β immune complex exists in the brain and hypothesized that the PD1-PDL1-GSK3β axis plays a vital role in tau hyperphosphorylation in AD. We found that the application of anti-PD1 blocking antibody reduced tau hyperphosphorylation and improved the memory ability of 5×FAD mice. Overall, our study highlights an important role of PD1 regulation in the treatment of human AD and provides a classic theoretical explanation for the immunotherapy of AD.

## Materials and Methods

### Materials

Anti-hamster IgG was obtained from SouthernBiotech, and anti-PD1 blocking antibody (G4) and anti-PDL1 antibody (10B5) were produced in-house. Detailed information on the antibodies used in the present study is presented in Supplementary Table S1.

### Animals

For this study, we used PD1 knockout (KO) mice, which has been described previously (Yao et al., 2009), APP/PS1 mice (obtained from GemPharmatech Co., Ltd.), 5×FAD mice, which has been described previously (An et al., 2019; Zheng et al., 2021), and their age-matched C57BL/6 wild-type (WT) mice. The age of PD1 KO, APP/PS1, and 5×FAD mice ranged from 2–3, 9–12, and 9–10 months, respectively. The experiments involving the mice were approved by the Experimental Animal Ethics Committee of Fujian Medical University (FJMU IACUC 2018-034).

### Intracerebroventricular Delivery of Aβ1-42 and anti-Programmed Cell Death-Receptor 1 Dosing Program

Synthetic β-amyloid (1–42) peptides (corresponding to the human Aβ sequence) were provided by China Peptides Co., Ltd. The Aβ1-42 powder was dissolved in sterile saline solution to a concentration of 1 μg/μl. The solution was then aged for 96 h at 37°C. Aβ aggregates or the corresponding vehicle (4 μl/5 min/mouse) was injected into the lateral ventricle (i.c.v.) using a microsyringe at the following position: −0.2 mm anteroposterior (AP), +1.0 mm mediolateral (ML), and −2.4 mm dorsoventral (DV) relative to the bregma (Amin et al., 2017; Wu et al., 2018). The control group mice received the first injection of hamster IgG (0.30 mg/mice) 3 days after the surgical procedures and the second injection 7 days after surgery. The Aβ1-42 + anti-PD1 group was injected with the PD1-blocking antibody following the same dosing program. Twelve days after surgery, all mice were sacrificed and the brain tissue was sampled for analysis (Zhang et al., 2020).

### Behavioral Testing

The dosing program of 5×FAD transgenic mice was based on a previous study (Rosenzweig et al., 2019). The mice were injected (i.p., 10 mg/kg) twice with anti-PD1 blocking antibody or anti-hamster IgG at a 3-day interval. The behavioral tests were conducted after 1 month, and all mice were sacrificed a month after the behavioral test. The Morris water maze (MWM) was performed to evaluate the spatial learning and memory ability of 5×FAD mice, as previously described (Qi et al., 2016; An et al., 2019). Briefly, a hidden escape platform (diameter: 100 mm, height: 230 mm) was placed at the center of one quadrant and 15 mm beneath the surface water in a pool of diameter 1,200 mm. Non-fat milk powder was added to turn the water opaque, and the temperature was maintained at 23–25°C. The behavioral test consisted of training for 5 days and a probe trial on day 6. During training, all mice were subjected to four trials per day, and the inter-trial interval was at least 10 min. For each trial, a mouse was released into warm water from the selected starting locations and allowed to locate the hidden platform within 1 min. The probe test was conducted to evaluate spatial memory ability on day 6. Mean escape latency, swimming speed, and number of platform crossing were analyzed using a computer equipped with Morris 2.8.1 software provided by Mobile Datum Co. (China).

### Tissue Preparation and Immunohistochemical Detection

For immunohistochemical evaluation of the mouse brain tissues, the mice were sacrificed and their brains were harvested, postfixed in 4% buffered paraformaldehyde overnight, embedded in paraffin, and cut into sections. Xylene was used to deparaffinize the paraffin-embedded tissue sections. The resulting sections were rehydrated with a gradient series of alcohol, and antigen retrieval was performed with citric buffer at 120°C for 10 min. To eliminate the influence of endogenous peroxidase and protein, 3% H_2_O_2_ and 10% fetal bovine serum (FBS) were successively added on the sections. Subsequently, the sections were incubated with the appropriate concentration of primary antibody overnight at 4°C, and then with HRP-conjugated secondary antibody for 30 min at room temperature. 3,3ʹ-Diaminobenzidine was added on the sections under a microscope and allowed to react for 2–5 min; the reaction was then stopped by adding H_2_O. The sections were then stained with hematoxylin and dehydrated with a gradient series of alcohol. After permeabilization with xylene, the sections were covered with permanent mounting medium. Positive staining was detected and calculated using Image-Pro Plus software for Windows operating system.

### Immunoblotting

Mouse hippocampal extracts were prepared by homogenizing the tissue in ice-cold RIPA lysis buffer supplemented with 1% (v/v) phenylmethanesulfonyl fluoride, as previously described (Gan et al., 2021). Boiled protein samples were subjected to SDS-PAGE, followed by semi-dry film transfer of the proteins onto polyvinylidene fluoride membranes. The membranes were blocked for 2 h with 10% (w/v) non-fat milk, and then probed with different antibodies overnight and HRP-conjugated secondary antibodies the next day. Various immunocomplexes were detected using the ChemiDoc XRS + system (BioRad).

### Coimmunoprecipitation

The brains of WT C57BL/6 mice were harvested and homogenized with a lysis buffer (Beyotime Biotechnology). Nonspecific IgG (Beyotime Biotechnology) or PDL1 antibody was added to the lysates, which were then incubated for 3–4 h. Thereafter, 40 μl of protein A/G beads (Santa Cruz Biotechnology) was added to the lysates and incubated for another 1–2 h at 4°C. The precipitates were washed with washing buffer at least five times and then separated by 10% SDS-PAGE for immunoblotting.

### Cell Culture

SH-SY5Y and SH-SY5Y-APP cell lines were provided by Professor Tae Ho Lee; these cell lines have been described elsewhere (Kim et al., 2016; Chen et al., 2020). The cells were cultured in Hyclone DME-F12 supplemented with FBS (10% v/v).

### Flow Cytometry

The cells were harvested and incubated with different primary antibodies for 30 min at 4°C, and then washed with PBS (1% FBS). The samples were analyzed using FACSVerse, and the data were analyzed using FlowJo software. APC-conjugated PD1 antibody and APC-conjugated PDL1 antibody were purchased from Biolegend.

### Statistical Analysis

All data are presented as mean ± SD and analyzed using GraphPad Prism version 8.0 software for statistical analysis. A one-way or two-way analysis of variance followed by Dunnett’s *post-hoc* test when appropriate or paired Student’s *t*-test was used to calculate statistical significance.

## Results

### Programmed Cell Death-Receptor 1 and Programmed Cell Death Receptor Ligand 1 Levels Were Elevated in Alzheimer’s Disease

**FIGURE 1:**
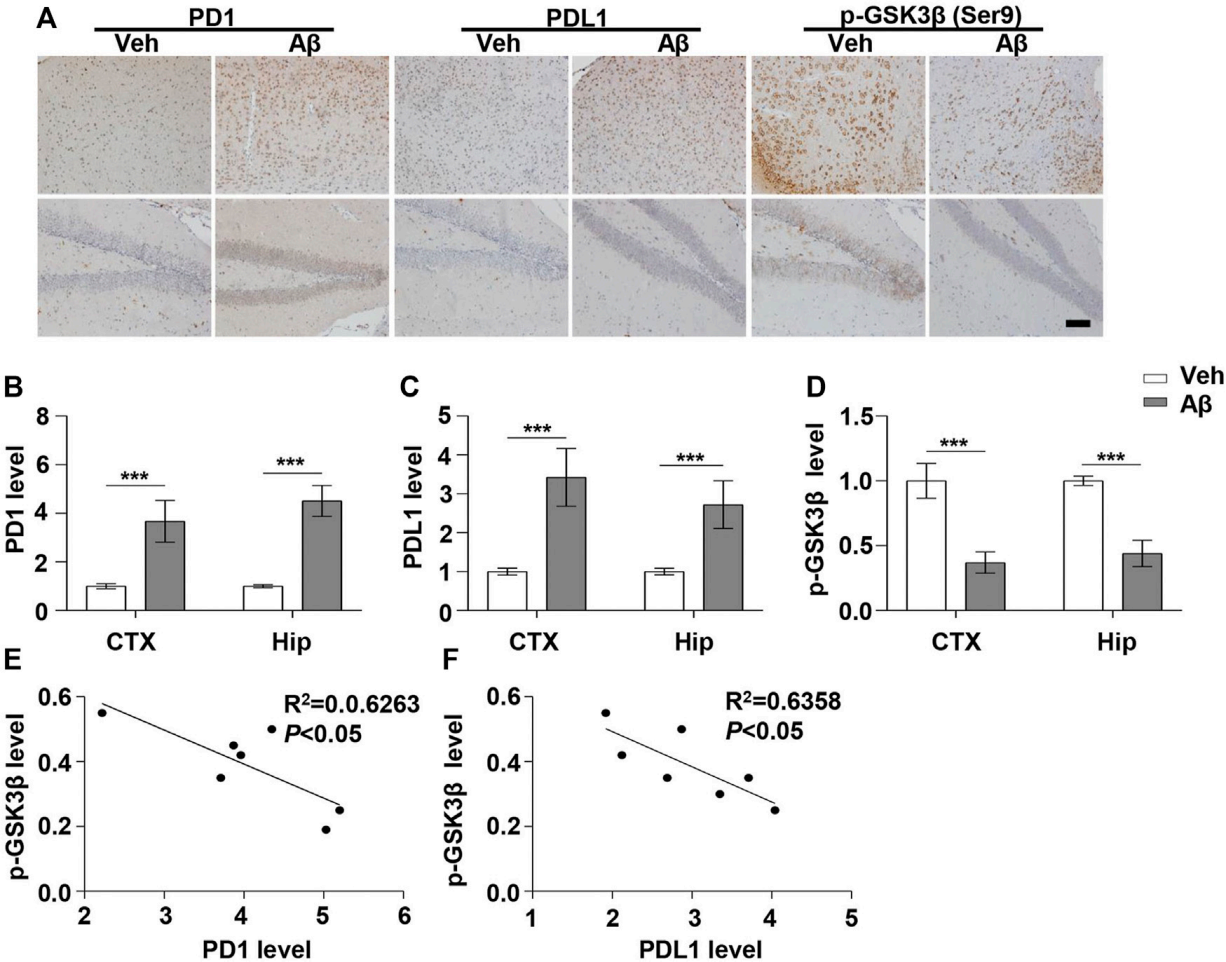
PD1 and PDL1 levels were elevated in the mouse brain post Aβ42 insult. Aβ42 was administered to WT (C57BL/6, female/male, aged 2–3 months, n = 7/group) mice, and after 12 days, the brain tissues were harvested. **(A–D)** Immunohistochemistry analysis using the anti-PD1, anti-PDL1, and p-GSK3β (Ser9) antibodies was performed with paraffin-embedded brain tissue sections from the vehicle and Aβ42-treated mice. ***p < 0.001; paired *t*-test, scale bar = 100 μm. **(E,F)** Correlation between the hippocampal p-GSK3β (Ser9) level on the *Y*-axis and the corresponding PD1 or PDL1 level on the *X*-axis (Pearson correlation coefficient, R^2^ = 0.6263, *p* < 0.05, or R^2^ = 0.6358, *p* < 0.05, respectively).

**FIGURE 2:**
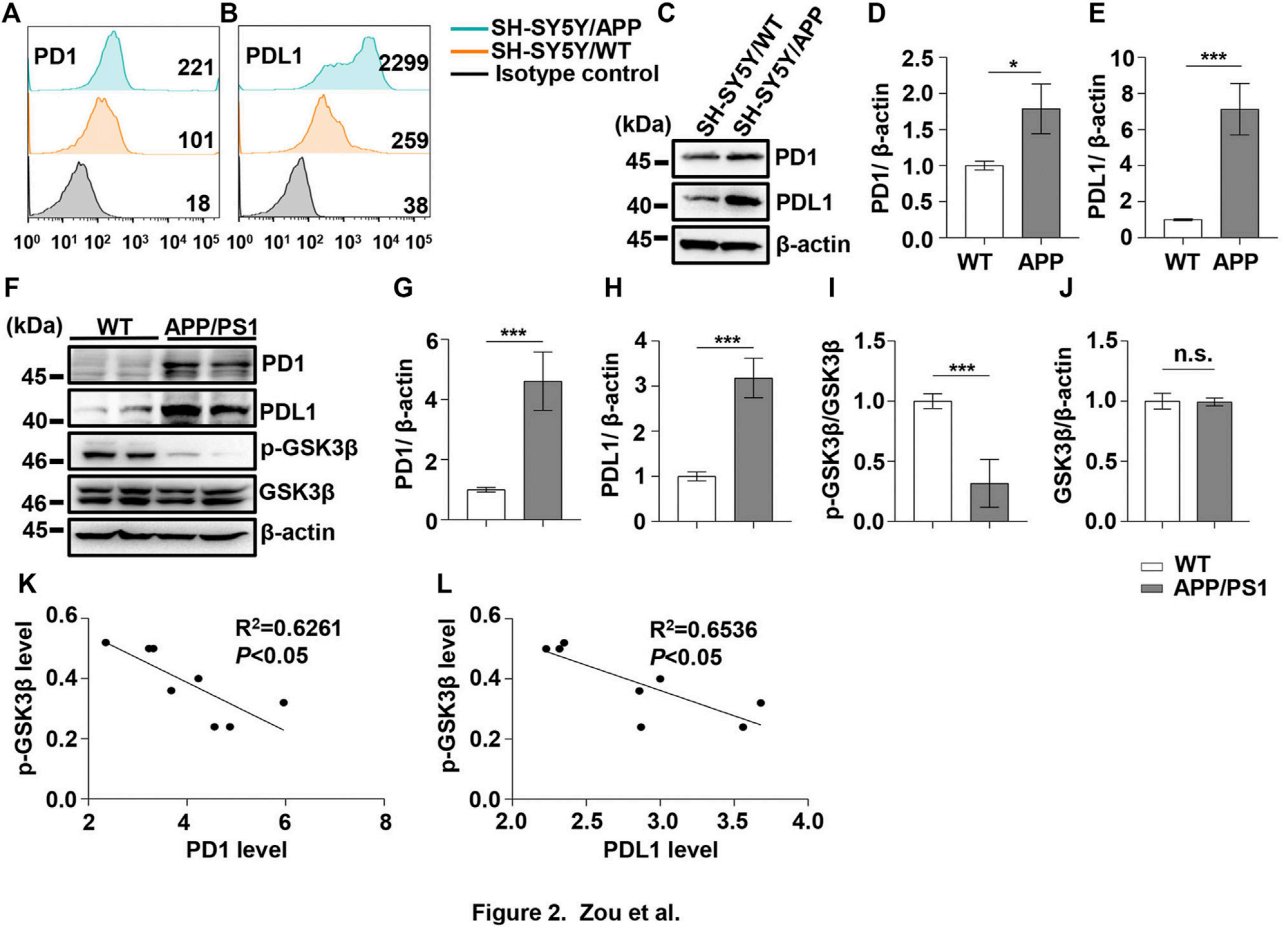
PD1 and PDL1 levels were elevated in SH-SY5Y-APP cell line and the APP/PS1 mouse brain. Cultured SH-SY5Y and SH-SY5Y-APP cells were collected for flow cytometry using the anti-PD1 **(A)** and anti-PDL1 antibodies **(B)**. **(C–E)** SH-SY5Y and SH-SY5Y-APP cell lysates were subjected to western blotting with the anti-β-actin, anti-PD1, or anti-PDL1 antibodies (***p < 0.001, *p < 0.05; paired *t*-test). WT and APP/PS1 mice (male and female, average age 9–12 months, n = 8/group) were sacrificed and the brain tissues were harvested. **(F–J)** Hippocampal tissue lysates were subjected to western blotting with the anti-β-actin, anti-PD1, anti-PDL1, p-GSK3β (Ser9), or GSK3β antibodies (***p < 0.001; paired *t*-test). **(K,L)** Correlation between the hippocampal p-GSK3β (Ser9) level on the *Y*-axis and the corresponding PD1 or PDL1 level on the *X*-axis (Pearson correlation coefficient, R^2^ = 0.6261, *p* < 0.05, or R^2^ = 0.6536, *p* < 0.05, respectively). n.s., *p* > 0.05. Data are representative of three independent experiments.

**FIGURE 3:**
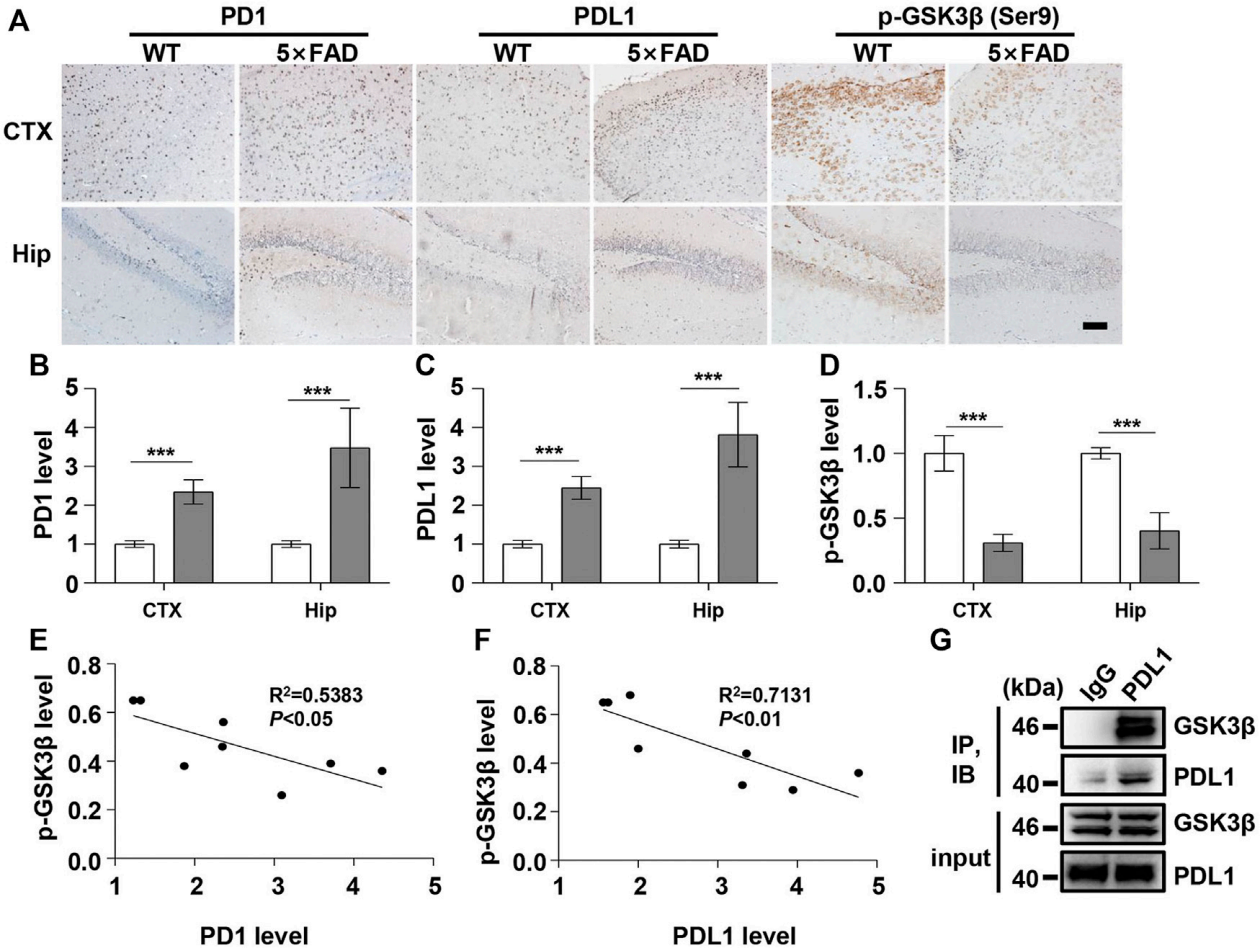
PD1 and PDL1 levels were elevated in the brain of 5×FAD mice. Male WT and 5×FAD mice (average age 9–10 months, n = 8/group) were sacrificed and their brain tissues were harvested. **(A–D)** Immunohistochemistry analysis using the anti-PD1, anti-PDL1 antibody, and p-GSK3β (Ser9) antibodies was performed with paraffin-embedded brain sections from WT and 5×FAD mice. ***p < 0.001; paired *t*-test, scale bar = 100 μm. **(E,F)** Correlation between the hippocampal p-GSK3β (Ser9) level on the *Y*-axis and the corresponding PD1 or PDL1 level on the *X*-axis (R^2^ = 0.5383, *p* < 0.05, or R^2^ = 0.7131, *p* < 0.01, respectively, Pearson correlation coefficient). **(G)** WT mouse brain lysates were precipitated with nonspecific IgG or anti-PDL1 antibodies and probed with anti-GSK3β.

Under normal physiological conditions, human primary neuroimmune cells express very low levels of PD1/PDL1, but neuronal PD1/PDL1 can be immediately induced by drug abuse or non-toxic doses of alcohol to contribute to neuroinflammation and neurodegeneration (Mishra et al., 2015; Mishra et al., 2020). The AD model generated by an intracerebroventricular injection of Aβ is commonly used for the following reasons: convenience and high cost-performance ratio. As shown in Figures 1A–C, the brain PD1 and PDL1 signals increased after Aβ42 insult compared with those after vehicle administration. SH-SY5Y cells stably overexpressing human APP have been widely used as an *in vitro* model to mimic AD pathology. We used flow cytometry and western blotting to compare the expression of PD1 and PDL1 between SH-SY5Y and SH-SY5Y-APP cells. There was an increase in the expression of PD1 and PDL1 in SH-SY5Y-APP cells, as shown in Figures 2A–E. Moreover, we performed western blotting and immunohistochemistry to detect the expression of PD1 and PDL1 in the brain of APP/PS1 and 5×FAD mice, respectively. The hippocampal levels of both PD1 and PDL1 were increased in APP/PS1 mice compared with those in the age-matched WT mice (Figures 2F–H). The PD1 and PDL1 levels were increased in a wide range of brain regions, including the cortex and hippocampus, in 5×FAD mice compared with those in the age matched WT mice (Figures 3A–C). These results suggest that PD1 and PDL1 are upregulated under AD conditions *in vitro* and *in vivo*.

### Glycogen Synthase Kinase 3 Beta Is a Suitable Downstream Target Molecule of Programmed Cell Death-Receptor 1/Programmed Cell Death Receptor Ligand 1 in the Central Nervous System

The hyperphosphorylation of tau, especially Thr231, which is a phosphorylation site of tau, mainly mediated by GSK3β (Israel et al., 2012; Moszczynski et al., 2015), peptidyl-prolyl *cis-trans* isomerase NIMA-interacting 1 (Kimura et al., 2013; Kim et al., 2021), cyclin-dependent protein kinase 5 (CDK5) (Crespo-Biel et al., 2007), and death-associated protein kinase 1 (DAPK1) (Kim et al., 2014; Chen et al., 2020), significantly decreases post anti-PD1 antibody treatment (Rosenzweig et al., 2019). Therefore, we sought to determine whether treatment with PD1-blocking antibodies affects the activities of some key tau kinases in the AD brain. We analyzed GSK3β as a downstream target molecule of PD1/PDL1 based on the following findings: 1) PDL1 interacts with GSK3β in tumor cells and GSK3β regulates PD1 level in CTL (Li et al., 2016); 2) GSK3β phosphorylates tau mainly at Thr231, Ser262, and Ser396 in the AD brain; and 3) GSK3β activity is closely associated with learning/memory impairment in AD. As low phosphorylated GSK3β (p-GSK3β) (Ser9) levels have been widely reported in various AD mouse models, such as APP/PS1 and 5×FAD (Crouch et al., 2009; Wang et al., 2019), GSK3β is considered the most important molecule in AD pathophysiology and a pivotal marker for neurodegeneration in AD (Takashima, 2006; Lauretti et al., 2020). In our study, the p-GSK3β (Ser9) level significantly decreased after Aβ insult (Figures 1A,D) and inversely correlated with PD1 or PDL1 expression, based on Pearson’s correlation efficient (Figures 1E,F; R^2^ = 0.6263, *p* = 0.0340 and R^2^ = 0.6358, *p* = 0.0317, respectively). To further verify the relationship between GSK3β and PD1 and PDL1, two other transgenic mice were used in the following experiments. In APP/PS1 mice, decreased p-GSK3β (Ser9) level (Figures 2F,I,J) inversely correlated with the PD1 or PDL1 level (Figures 2K,L; R^2^ = 0.6261, *p* = 0.0193 and R^2^ = 0.6536, *p* = 0.0151, respectively). Moreover, activated GSK3β level in 5×FAD mice was significantly increased compared to that in WT littermates (Figures 3A,D). Furthermore, as shown in Figures 3E,F, an inverse correlation was observed between PD1 or PDL1 expression and p-GSK3β (Ser9) level (R^2^ = 0.5383, *p* = 0.0383 and R^2^ = 0.7131, *p* = 0.0083, respectively). Moreover, co-immunoprecipitation and double immunofluorescence assay revealed the PDL1/GSK3β immune complex in the brain and in SH-SY5Y-APP cells, respectively (Figure 3G and Supplementary Figure S1), suggesting that GSK3β might act directly downstream of the PD1/PDL1 axis. These results demonstrate that the PD1/PDL1 axis may be involved in AD pathology through GSK3β.

### Programmed Cell Death-Receptor 1 Ablation Decreased Glycogen Synthase Kinase 3 Beta Activity and Tau Hyperphosphorylation Induced by Aβ42 Exposure

**FIGURE 4:**
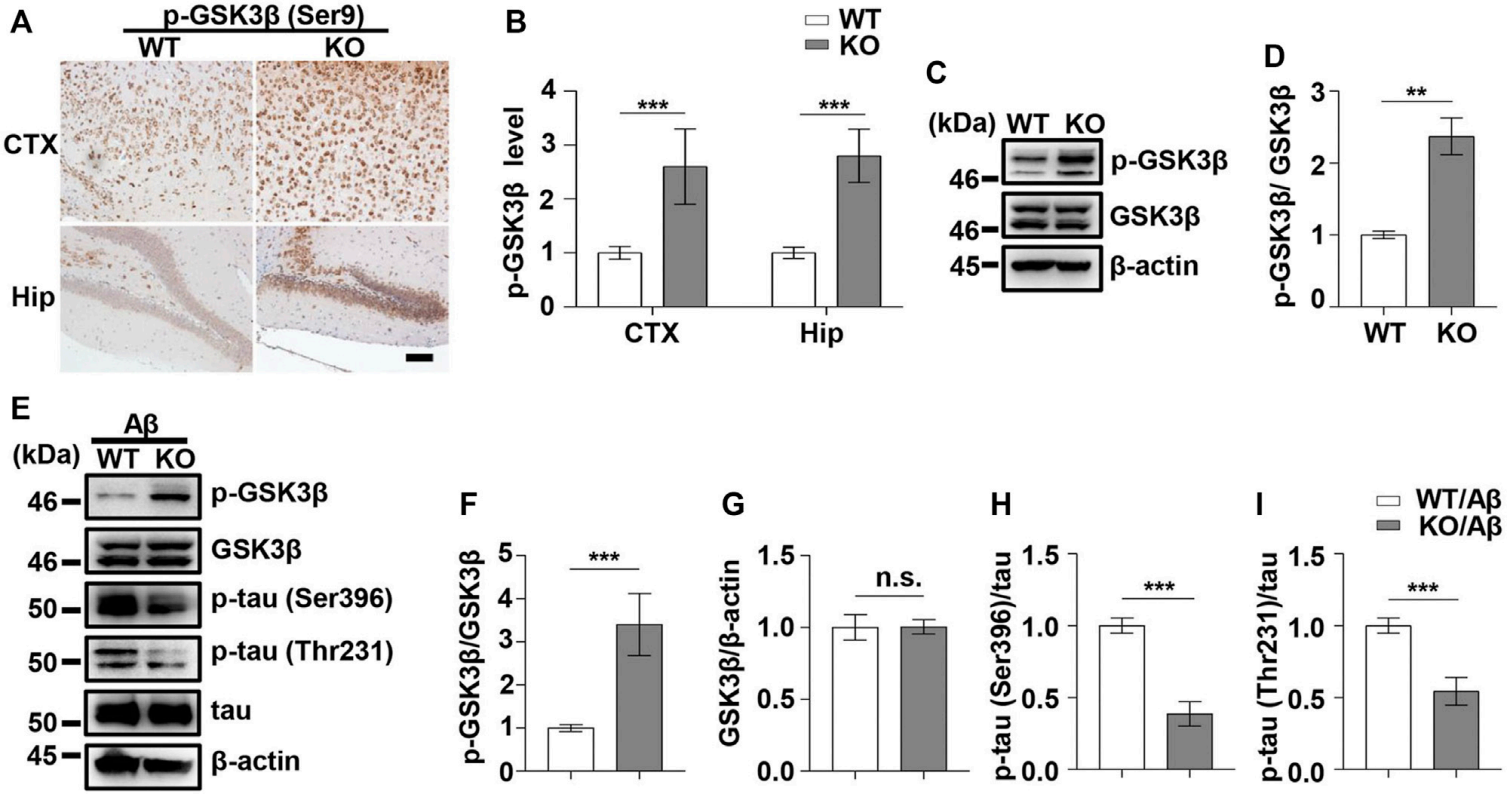
PD1 deficiency reduced GSK3β activity and tau hyperphosphorylation induced by Aβ42 insult. WT and PD1-deficient mice (C57BL/6, female and male, aged 2–3 months, n = 8/group) were anesthetized and sacrificed, and the brain tissues were harvested for analysis. **(A,B)** Immunohistochemistry analysis using the anti-pGSK3β (Ser9) antibody was performed with paraffin-embedded brain tissue sections from WT and PD1 KO mice (***p < 0.001; paired *t*-test, scale bar = 100 μm). **(C,D)** Hippocampal tissue lysates were subjected to western blotting with the anti-β-actin, anti-pGSK3β (Ser9), and anti-GSK3β antibodies (***p < 0.001, **p < 0.01; paired *t*-test). WT and PD1 KO (C57BL/6, female and male, aged 2–3 months, n = 8/group) mice were treated with Aβ42, and after 12 days, the brain tissues were harvested. **(E–I)** Hippocampal tissue lysates were subjected to western blotting with the anti-β-actin, anti-pGSK3β (Ser9), anti-GSK3β, anti-p-tau (Thr231), anti-p-tau (Ser396), and anti-tau antibodies (***p < 0.001). n.s., *p* > 0.05.

As PD1 is considered a regulator of GSK3β phosphorylation in different AD models, we aimed to clarify whether the upregulation of GSK3β activity in AD conditions is associated with PD1. PD1 deficiency tends to increase pS473-AKT level in normal Kupffer cells and restores AKT activation after murine polymicrobial sepsis attack, suggesting that PD1 KO protects cells from injury stimuli (Wang et al., 2016). Unexpectedly, in the present study, the phosphorylation of GSK3β at Ser9 in the brain of PD1 KO mice was significantly increased compared with that in age-matched WT mice, as shown in Figures 4A–D, suggesting that PD1 deficiency downregulates the activity of GSK3β under normal physiological conditions. We also investigated the effect of PD1 deficiency on GSK3β activity and tau hyperphosphorylation (p-tau Thr231 and Ser396) after an intracerebroventricular Aβ42 insult. Consistent with previous results, the pSer9 level in GSK3β decreased significantly after Aβ exposure. Aβ-treated PD1 KO mice showed a significant increase in the pSer9 level and decrease in the p-tau Thr231 and Ser396 levels compared with the control mice (Figures 4E–I). These results indicate that PD1 is an important regulator of GSK3β activity under normal physiological and AD conditions.

### Programmed Cell Death-Receptor 1 Blockade Reduced Glycogen Synthase Kinase 3 Beta Activity and Tau Hyperphosphorylation and Improved Memory in Alzheimer’s Disease Mice Models

**FIGURE 5:**
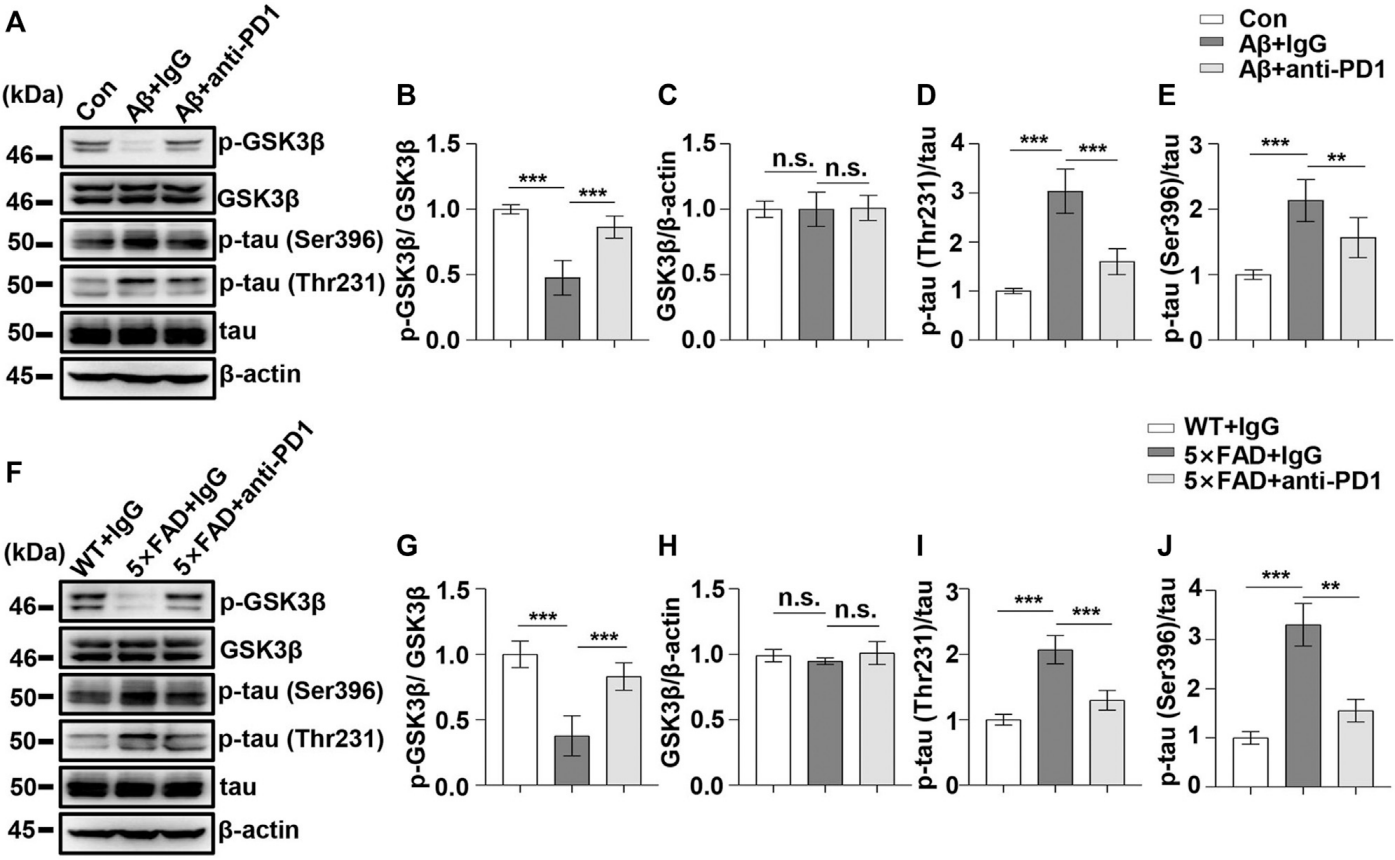
PD1 blockade reduced GSK3β activity and tau hyperphosphorylation in AD mouse models. WT mice (C57BL/6, male and female, aged 2–3 months, n = 10/group) were treated with Aβ42, and then with anti-PD1 or control hamster IgG; after 12 days, the brain tissues were harvested. **(A–E)** PD1 blockade reduced GSK3β activity and tau hyperphosphorylation. Hippocampal tissue lysates were subjected to western blotting with the anti-β-actin, anti-pGSK3β (Ser9), anti-GSK3β, anti-p-tau (Thr231), anti-p-tau (Ser396), and anti-tau antibodies (***p < 0.001, **p < 0.01, one-way ANOVA followed by Dunnett’s *post-hoc* test). Male 5×FAD mice (average age 9–10 months, n = 10/group) were administered either anti-PD1 antibody or control hamster IgG. The brain tissues were harvested 1 month after MWM. **(F–J)** Hippocampal tissue lysates were subjected to western blotting with the anti-β-actin, anti-pGSK3β (Ser9), anti-GSK3β, anti-p-tau (Thr231), anti-p-tau (Ser396), and anti-tau antibodies (***p < 0.001, **p < 0.01, one-way ANOVA followed by Dunnett’s *post-hoc* test). n.s., *p* > 0.05. Data are representative of three independent experiments.

Next, we examined the effect of PD1-blocking antibody on Aβ-induced GSK3β activation and tau hyperphosphorylation. PD1-blocking antibody increased the expression of phosphorylated GSK3β at Ser9 and reduced the p-tau Thr231 and Ser396 levels after Aβ42 administration compared with those in the control, as shown in Figures 5A–E. Similarly, we used 5×FAD mice to investigate the protective effect of PD1-blocking antibody. We examined pSer9-GSK3β and total GSK3β levels in the hippocampus of 5×FAD mice by western blotting. The results showed a significant increase in pSer9-GSK3β rather than the total GSK3β level in the brain of anti-PD1-treated 5×FAD mice compared with that in the IgG-treated mice (Figures 5F–H), suggesting that GSK3β activity decreased after PD1 blockade. We further explored the effects of immunotherapy on tau hyperphosphorylation. Consistent with the results of a previous study (Rosenzweig et al., 2019), we found that both p-tau Thr231 and Ser396 levels decreased after intervention with PD1-blocking antibody (Figures 5F,I,J). These results indicate that blocking the PD1/PDL1 axis significantly reduces GSK3β activity and tau hyperphosphorylation in different AD models.

**FIGURE 6:**
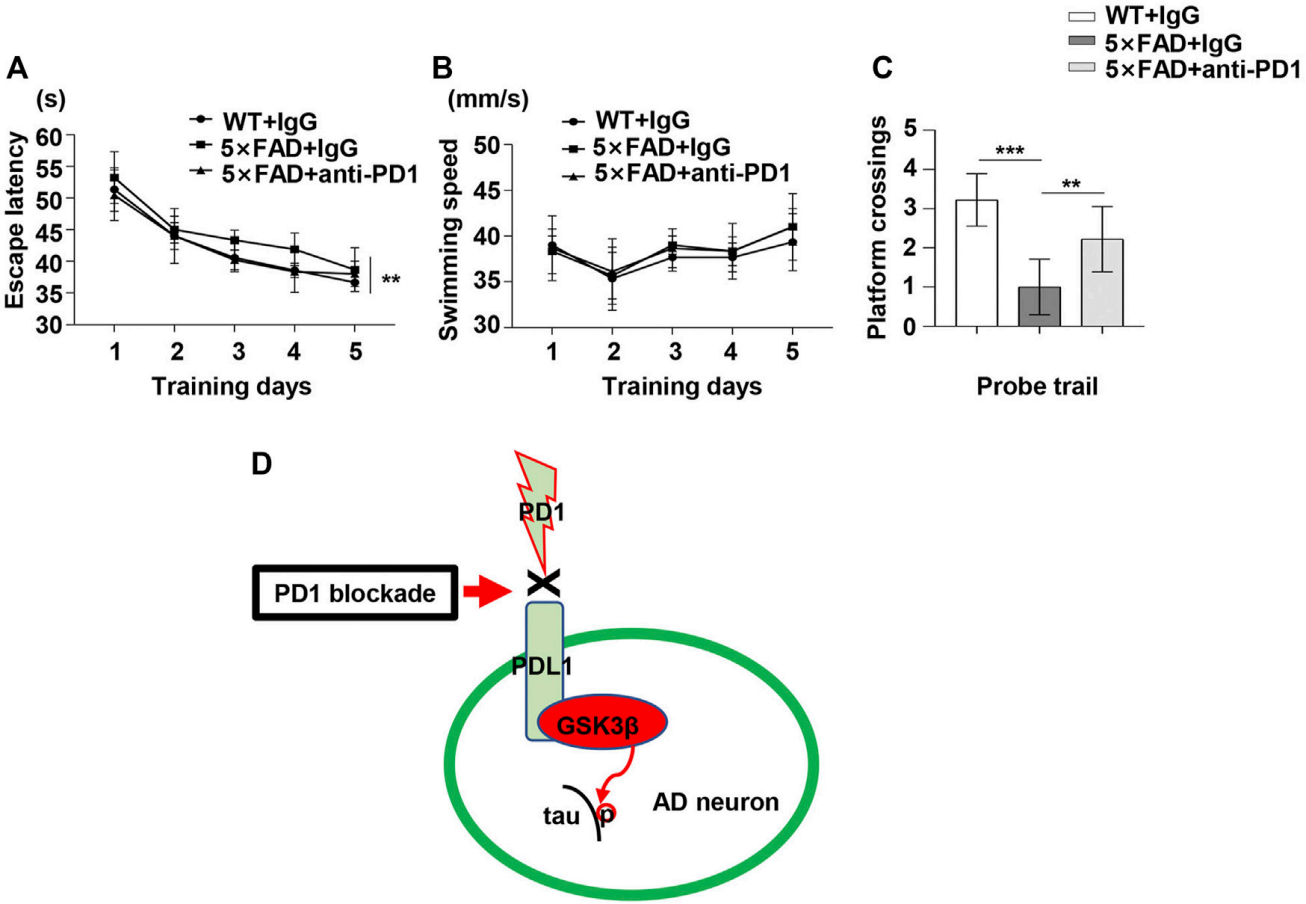
PD1 blockade improved memory in 5×FAD mice. Male 5×FAD mice (average age 9–10 months, n = 9/group) were administered either anti-PD1 monoclonal antibody or control hamster IgG. MWM was performed 1 month later. **(A–C)** PD1 blockade significantly improved behavioral performance compared with hamster IgG treatment. **(A)** Escape latency and **(B)** swimming speed during the training trial (**p < 0.01, two-way ANOVA followed by Dunnett’s *post-hoc* test). **(C)** Platform crossing during the probe trial (***p < 0.001, **p < 0.01, one-way ANOVA followed by Dunnett’s *post-hoc* test). **(D)** The proposed role of the PD1/PDL1 axis in the regulation of GSK3β activity is summarized in a schematic diagram. Under AD conditions, the PD1-PDL1 pathway is activated, leading to increased activity of the effector GSK3β. PD1 deficiency or PD1 antibody blocks the PD1/PDL1 axis, ultimately decreases GSK3β activity and ameliorates tau hyperphosphorylation in the AD brain.

To determine the potential role of PD1 blockade in the learning and memory abilities in 5×FAD mice, hippocampus-dependent cognitive performance was evaluated using the MWM 1 month after the antibody treatment. As shown in Figure 6A, the 5×FAD mice presented an increase in the mean escape latency from the hidden platform compared with the controls (*p* < 0.01). Moreover, the average escape latency of the anti-PD1+5×FAD group was significantly lower than that of the IgG+5×FAD group (*p* < 0.05). However, there was no significant difference in the mean swimming speed during the training phase among the three groups (Figure 6B, *p* > 0.05). In the probe trial, the number of platform crossing was determined for 1 min on day 6 of the test. As expected, 5×FAD mice treated with control IgG had fewer platform crossings than the normal control subjects, and this was reversed by PD1-blocking antibody treatment (Figure 6C). These results suggest that the application of PD1-blocking antibody alleviates impaired cognitive performance in 5×FAD mice.

## Discussion

We aimed to develop a suitable model wherein PD1 regulates GSK3β activity, which may be related to tau hyperphosphorylation and cognitive dysfunction in AD (Figure 6D). Inhibiting PD1 expression by KO or blocking PD1 with a specific antibody significantly reversed GSK3β activity and tau hyperphosphorylation. Thus, we identified that PD1 is a critical regulator of GSK3β and that it could possibly bridge classic AD pathogenic theories and the recent immunotherapy strategies.

PD1/PDL1 checkpoint blockade exerts a potent protective effect against cognitive impairment and tau hyperphosphorylation in various mouse models of AD (Baruch et al., 2016; Rosenzweig et al., 2019) and stroke (Ren et al., 2011; Bodhankar et al., 2015); but there are also some contradictory conclusions (Latta-Mahieu et al., 2018; Lin et al., 2019). It is impressive that tau hyperphosphorylation and behavioral memory impairment can be rescued by a simple treatment with antibodies, but the underlying mechanism is not yet clear. Here, we elucidated the key role of PD1/PDL1 in various mouse models. It has been reported that the level of PD1 rather than PDL1 is increased in an experimental model of prion disease, a classic murine model of chronic neurodegeneration (Obst et al., 2018). Whereas, the expression of PD1 and PDL1 is upregulated after transient cerebral artery occlusion treatment, representing an experimental model of stroke (Ren et al., 2011), suggesting that the PD1/PDL1 axis may be closely involved in diseases of the central nervous system. PD1-blocking antibody exerted a strong AD therapeutic effect in aged (9–10 months old) 5×FAD mice (Baruch et al., 2016). However, it was still unclear whether PD1 and PDL1 are expressed in the brain of AD mice. In this milieu, to the best of our knowledge, this study is the first to demonstrate that the PD1 and PDL1 levels are increased in several AD mouse models and in SH-SY5Y cells overexpressing human APP695. It is interesting that PD1/PDL1 can be upregulated by APP overexpression, suggesting a critical role for APP in PD1/PDL1 expression. Moreover, the upregulation of PD1 or PDL1 in the AD mouse brain positively correlated with GSK3β activity, suggesting a relationship between PD1/PDL1 and GSK3β. To verify the AD pathological changes that are mediated by PD1, we chose GSK3β as the downstream target of PD1/PDL1 because the PDL1/GSK3β complex exists in tumor cells and the brain. Unexpectedly, the GSK3β activity decreased in PD1 KO mice compared with that in age-matched WT mice. Mechanistically, intracerebroventricular administration of Aβ activates GSK3β through PD1. Similarly, in 5×FAD mice, PD1 blockade downregulated the GSK3β activity. The formation of intracellular neurofibrillary tangles because of hyperphosphorylation of tau is another pathological change in AD. Tau hyperphosphorylation in different sites is mediated by different kinases, including CDK5, GSK3β, and DAPK1. In this study, PD1 blockade significantly reduced the levels of p-tau Thr231 and Ser396 in an Aβ-induced AD mouse model and 5×FAD mouse model. This may be closely related to GSK3β as Thr231 and Ser396 are considered the main phosphorylation sites for GSK3β in AD. However, our study has some limitations. We investigated the therapeutic effects post PD1 blockade and the underlying mechanism in only amyloid mouse models. As changes in the cognitive function after PD1/PDL1 blockade in both amyloid and tauopathy models have been confirmed (Baruch et al., 2016; Rosenzweig et al., 2019), we will use diverse models such as 3×Tg-AD mice and htau transgenic mice to further verify the PD1-PDL1-GSK3β-tau axis in the future. On the contrary, behavioral changes in both 5×FAD and DM-hTAU mice post anti-PD1/PDL1 therapy could involve other mechanisms besides a reduction in GSK3β activity, such as recruitment of monocyte-derived macrophages to the central nervous system to evoke a systemic immune response (Baruch et al., 2016; Rosenzweig et al., 2019). Future studies should focus on the mechanism of anti-PD1/PDL1 therapy against AD.

In conclusion, our study lays a solid theoretical and experimental basis for the immunotherapy of AD, and immune checkpoint inhibitors are expected to become potent tools for the treatment of AD.

## Data Availability

The datasets presented in this study can be found in online repositories. The names of the repository/repositories and accession number(s) can be found in the article/Supplementary Material.

## Abbreviations

PD1, programmed cell death-receptor 1; PDL1, programmed cell death receptor ligand 1; GSK3β, glycogen synthase kinase 3 beta; AD, Alzheimer’s disease; FBS, fetal bovine serum.

## References

[B1] Amin F. U. Shah S. A. Kim M. O. (2017). Vanillic Acid Attenuates Aβ1-42-Induced Oxidative Stress and Cognitive Impairment in Mice. Sci. Rep. 7, 40753. 10.1038/srep40753 28098243 PMC5241654

[B2] An J. Zhou Y. Zhang M. Xie Y. Ke S. Liu L. (2019). Exenatide Alleviates Mitochondrial Dysfunction and Cognitive Impairment in the 5×FAD Mouse Model of Alzheimer's Disease. Behav. Brain Res. 370, 111932. 10.1016/j.bbr.2019.111932 31082410

[B3] Baruch K. Deczkowska A. Rosenzweig N. Tsitsou-Kampeli A. Sharif A. M. Matcovitch-Natan O. (2016). PD-1 Immune Checkpoint Blockade Reduces Pathology and Improves Memory in Mouse Models of Alzheimer's Disease. Nat. Med. 22 (2), 135–137. 10.1038/nm.4022 26779813

[B4] Berger T. Lee H. Young A. H. Aarsland D. Thuret S. (2020). Adult Hippocampal Neurogenesis in Major Depressive Disorder and Alzheimer's Disease. Trends Mol. Med. 26 (9), 803–818. 10.1016/j.molmed.2020.03.010 32418723

[B5] Billingsley M. L. Kincaid R. L. (1997). Regulated Phosphorylation and Dephosphorylation of Tau Protein: Effects on Microtubule Interaction, Intracellular Trafficking and Neurodegeneration. Biochem. J. 323 (Pt 3), 577–591. 10.1042/bj3230577 9169588 PMC1218358

[B6] Bloom G. S. (2014). Amyloid-β and Tau: the Trigger and Bullet in Alzheimer Disease Pathogenesis. JAMA Neurol. 71 (4), 505–508. 10.1001/jamaneurol.2013.5847 24493463 PMC12908160

[B7] Bodhankar S. Chen Y. Lapato A. Dotson A. L. Wang J. Vandenbark A. A. (2015). PD-L1 Monoclonal Antibody Treats Ischemic Stroke by Controlling Central Nervous System Inflammation. Stroke 46 (10), 2926–2934. 10.1161/strokeaha.115.010592 26306753 PMC4589506

[B8] Brahmer J. R. Tykodi S. S. Chow L. Q. M. Hwu W.-J. Topalian S. L. Hwu P. (2012). Safety and Activity of Anti-PD-L1 Antibody in Patients with Advanced Cancer. N. Engl. J. Med. 366 (26), 2455–2465. 10.1056/NEJMoa1200694 22658128 PMC3563263

[B9] Chen D. Mei Y. Kim N. Lan G. Gan C. L. Fan F. (2020). Melatonin Directly Binds and Inhibits Death‐associated Protein Kinase 1 Function in Alzheimer's Disease. J. Pineal Res. 69 (2), e12665. 10.1111/jpi.12665 32358852 PMC7890046

[B10] Crespo-Biel N. Canudas A. M. Camins A. Pallàs M. (2007). Kainate Induces AKT, ERK and cdk5/GSK3β Pathway Deregulation, Phosphorylates Tau Protein in Mouse hippocampus. Neurochem. Int. 50 (2), 435–442. 10.1016/j.neuint.2006.10.002 17116346

[B11] Crouch P. J. Hung L. W. Adlard P. A. Cortes M. Lal V. Filiz G. (2009). Increasing Cu Bioavailability Inhibits A Oligomers and Tau Phosphorylation. Proc. Natl. Acad. Sci. 106 (2), 381–386. 10.1073/pnas.0809057106 19122148 PMC2626711

[B12] Fife B. T. Pauken K. E. (2011). The Role of the PD-1 Pathway in Autoimmunity and Peripheral Tolerance. Ann. N. Y Acad. Sci. 1217, 45–59. 10.1111/j.1749-6632.2010.05919.x 21276005

[B13] Gan C.-L. Zou Y. Xia Y. Zhang T. Chen D. Lan G. (2021). Inhibition of Death-Associated Protein Kinase 1 Protects against Epileptic Seizures in Mice. Int. J. Biol. Sci. 17 (9), 2356–2366. 10.7150/ijbs.59922 34239362 PMC8241737

[B14] Haass C. Selkoe D. J. (1993). Cellular Processing of β-amyloid Precursor Protein and the Genesis of Amyloid β-peptide. Cell 75 (6), 1039–1042. 10.1016/0092-8674(93)90312-e 8261505

[B15] Hanger D. P. Byers H. L. Wray S. Leung K.-Y. Saxton M. J. Seereeram A. (2007). Novel Phosphorylation Sites in Tau from Alzheimer Brain Support a Role for Casein Kinase 1 in Disease Pathogenesis. J. Biol. Chem. 282 (32), 23645–23654. 10.1074/jbc.M703269200 17562708

[B16] Ishida Y. Agata Y. Shibahara K. Honjo T. (1992). Induced Expression of PD-1, a Novel Member of the Immunoglobulin Gene Superfamily, upon Programmed Cell Death. EMBO J. 11 (11), 3887–3895. 10.1002/j.1460-2075.1992.tb05481.x 1396582 PMC556898

[B17] Israel M. A. Yuan S. H. Bardy C. Reyna S. M. Mu Y. Herrera C. (2012). Probing Sporadic and Familial Alzheimer's Disease Using Induced Pluripotent Stem Cells. Nature 482 (7384), 216–220. 10.1038/nature10821 22278060 PMC3338985

[B18] Jiang C. Wang Z. Donnelly C. R. Wang K. Andriessen A. S. Tao X. (2020). PD-1 Regulates GABAergic Neurotransmission and GABA-Mediated Analgesia and Anesthesia. iScience 23 (10), 101570. 10.1016/j.isci.2020.101570 33083737 PMC7530307

[B19] Kim B. M. You M.-H. Chen C.-H. Lee S. Hong Y. Hong Y. (2014). Death-associated Protein Kinase 1 Has a Critical Role in Aberrant Tau Protein Regulation and Function. Cell Death Dis. 5 (5), e1237. 10.1038/cddis.2014.216 24853415 PMC4047864

[B20] Kim B. M. You M.-H. Chen C.-H. Suh J. Tanzi R. E. Ho Lee T. (2016). Inhibition of Death-Associated Protein Kinase 1 Attenuates the Phosphorylation and Amyloidogenic Processing of Amyloid Precursor Protein. Hum. Mol. Genet. 25 (12), ddw114–2513. 10.1093/hmg/ddw114 PMC6086563 27094130

[B21] Kim N. Wang B. Koikawa K. Nezu Y. Qiu C. Lee T. H. (2021). Inhibition of Death-Associated Protein Kinase 1 Attenuates Cis P-Tau and Neurodegeneration in Traumatic Brain Injury. Prog. Neurobiol. 203, 102072. 10.1016/j.pneurobio.2021.102072 33979671 PMC8217320

[B22] Kimura T. Tsutsumi K. Taoka M. Saito T. Masuda-Suzukake M. Ishiguro K. (2013). Isomerase Pin1 Stimulates Dephosphorylation of Tau Protein at Cyclin-dependent Kinase (Cdk5)-dependent Alzheimer Phosphorylation Sites. J. Biol. Chem. 288 (11), 7968–7977. 10.1074/jbc.M112.433326 23362255 PMC3597833

[B23] Latta-Mahieu M. Elmer B. Bretteville A. Wang Y. Lopez-Grancha M. Goniot P. (2018). Systemic Immune-Checkpoint Blockade with Anti-PD1 Antibodies Does Not Alter Cerebral Amyloid-β burden in Several Amyloid Transgenic Mouse Models. Glia 66 (3), 492–504. 10.1002/glia.23260 29134678

[B24] Lauretti E. Dincer O. Praticò D. (2020). Glycogen Synthase Kinase-3 Signaling in Alzheimer's Disease. Biochim. Biophys. Acta (Bba) - Mol. Cel Res. 1867 (5), 118664. 10.1016/j.bbamcr.2020.118664 PMC7047718 32006534

[B25] Leroy K. Yilmaz Z. Brion J. P. (2007). Increased Level of Active GSK-3beta in Alzheimer's Disease and Accumulation in Argyrophilic Grains and in Neurones at Different Stages of Neurofibrillary Degeneration. Neuropathol. Appl. Neurobiol. 33 (1), 43–55. 10.1111/j.1365-2990.2006.00795.x 17239007

[B26] Leroy A. Landrieu I. Huvent I. Legrand D. Codeville B. Wieruszeski J.-M. (2010). Spectroscopic Studies of GSK3β Phosphorylation of the Neuronal Tau Protein and its Interaction with the N-Terminal Domain of Apolipoprotein E. J. Biol. Chem. 285 (43), 33435–33444. 10.1074/jbc.M110.149419 20679343 PMC2963357

[B27] Li T. Paudel H. K. (2006). Glycogen Synthase Kinase 3β Phosphorylates Alzheimer's Disease-specific Ser396 of Microtubule-Associated Protein Tau by a Sequential Mechanism. Biochemistry 45 (10), 3125–3133. 10.1021/bi051634r 16519507

[B28] Li T. Hawkes C. Qureshi H. Y. Kar S. Paudel H. K. (2006). Cyclin-Dependent Protein Kinase 5 Primes Microtubule-Associated Protein Tau Site-Specifically for Glycogen Synthase Kinase 3β. Biochemistry 45 (10), 3134–3145. 10.1021/bi051635j 16519508

[B29] Li C.-W. Lim S.-O. Xia W. Lee H.-H. Chan L.-C. Kuo C.-W. (2016). Glycosylation and Stabilization of Programmed Death Ligand-1 Suppresses T-Cell Activity. Nat. Commun. 7, 12632. 10.1038/ncomms12632 27572267 PMC5013604

[B30] Lin Y. Rajamohamedsait H. B. Sandusky-Beltran L. A. Gamallo-Lana B. Mar A. Sigurdsson E. M. (2019). Chronic PD-1 Checkpoint Blockade Does Not Affect Cognition or Promote Tau Clearance in a Tauopathy Mouse Model. Front. Aging Neurosci. 11, 377. 10.3389/fnagi.2019.00377 31992982 PMC6971044

[B31] Mishra V. Schuetz H. Haorah J. (2015). Differential Induction of PD-1/pd-L1 in Neuroimmune Cells by Drug of Abuse. Int. J. Physiol. Pathophysiol. Pharmacol. 7 (2), 87–97. 26330898 PMC4550211

[B32] Mishra V. Agas A. Schuetz H. Kalluru J. Haorah J. (2020). Alcohol Induces Programmed Death Receptor-1 and Programmed Death-Ligand-1 Differentially in Neuroimmune Cells. Alcohol 86, 65–74. 10.1016/j.alcohol.2020.03.009 32224220

[B33] Moszczynski A. J. Gohar M. Volkening K. Leystra-Lantz C. Strong W. Strong M. J. (2015). Thr175-phosphorylated Tau Induces Pathologic Fibril Formation via GSK3β-Mediated Phosphorylation of Thr231 *In Vitro* . Neurobiol. Aging 36 (3), 1590–1599. 10.1016/j.neurobiolaging.2014.12.001 25573097

[B34] Obst J. Mancuso R. Simon E. Gomez-Nicola D. (2018). PD-1 Deficiency Is Not Sufficient to Induce Myeloid Mobilization to the Brain or Alter the Inflammatory Profile during Chronic Neurodegeneration. Brain Behav. Immun. 73, 708–716. 10.1016/j.bbi.2018.08.006 30086399 PMC6191933

[B35] Peprah K. McCormack S. (2019). “CADTH Rapid Response Reports,” in Medical Cannabis for the Treatment of Dementia: A Review of Clinical Effectiveness and Guidelines (Ottawa (ON): Canadian Agency for Drugs and Technologies in Health). Canadian Agency for Drugs and Technologies in Health Copyright © 2019. 31525011

[B36] Qi L. Ke L. Liu X. Liao L. Ke S. Liu X. (2016). Subcutaneous Administration of Liraglutide Ameliorates Learning and Memory Impairment by Modulating Tau Hyperphosphorylation via the Glycogen Synthase Kinase-3β Pathway in an Amyloid β Protein Induced Alzheimer Disease Mouse Model. Eur. J. Pharmacol. 783, 23–32. 10.1016/j.ejphar.2016.04.052 27131827

[B37] Ren X. Akiyoshi K. Vandenbark A. A. Hurn P. D. Offner H. (2011). Programmed Death-1 Pathway Limits central Nervous System Inflammation and Neurologic Deficits in Murine Experimental Stroke. Stroke 42 (9), 2578–2583. 10.1161/strokeaha.111.613182 21737801 PMC3164218

[B38] Rosenzweig N. Dvir-Szternfeld R. Tsitsou-Kampeli A. Keren-Shaul H. Ben-Yehuda H. Weill-Raynal P. (2019). PD-1/PD-L1 Checkpoint Blockade Harnesses Monocyte-Derived Macrophages to Combat Cognitive Impairment in a Tauopathy Mouse Model. Nat. Commun. 10 (1), 465. 10.1038/s41467-019-08352-5 30692527 PMC6349941

[B39] Schulz D. Stancev I. Sorrentino A. Menevse A.-N. Beckhove P. Brockhoff G. (2019). Increased PD-L1 Expression in Radioresistant HNSCC Cell Lines after Irradiation Affects Cell Proliferation Due to Inactivation of GSK-3beta. Oncotarget 10 (5), 573–583. 10.18632/oncotarget.26542 30728908 PMC6355177

[B40] Schwartz M. Arad M. Ben-Yehuda H. (2019). Potential Immunotherapy for Alzheimer Disease and Age-Related Dementia. Dialogues Clin. Neurosci. 21 (1), 21–25. 10.31887/DCNS.2019.21.1/mschwartz 31607777 PMC6780363

[B41] Selkoe D. J. Hardy J. (2016). The Amyloid Hypothesis of Alzheimer's Disease at 25 Years. EMBO Mol. Med. 8 (6), 595–608. 10.15252/emmm.201606210 27025652 PMC4888851

[B42] Takashima A. Noguchi K. Michel G. Mercken M. Hoshi M. Ishiguro K. (1996a). Exposure of Rat Hippocampal Neurons to Amyloid β Peptide (25-35) Induces the Inactivation of Phosphatidyl Inositol-3 Kinase and the Activation of Tau Protein Kinase I/glycogen Synthase Kinase-3β. Neurosci. Lett. 203 (1), 33–36. 10.1016/0304-3940(95)12257-5 8742040

[B43] Takashima A. Sato M. Mercken M. Tanaka S. Kondo S. Honda T. (1996b). Localization of Alzheimer-Associated Presenilin 1 in Transfected COS-7 Cells. Biochem. Biophys. Res. Commun. 227 (2), 423–426. 10.1006/bbrc.1996.1523 8878531

[B44] Takashima A. (2006). GSK-3 Is Essential in the Pathogenesis of Alzheimer's Disease. J. Alzheimers Dis. 9 (3 Suppl. l), 309–317. 10.3233/jad-2006-9s335 16914869

[B45] Taylor A. Harker J. A. Chanthong K. Stevenson P. G. Zuniga E. I. Rudd C. E. (2016). Glycogen Synthase Kinase 3 Inactivation Drives T-Bet-Mediated Downregulation of Co-receptor PD-1 to Enhance CD8^+^ Cytolytic T Cell Responses. Immunity 44 (2), 274–286. 10.1016/j.immuni.2016.01.018 26885856 PMC4760122

[B46] Wang F. Huang X. Chung C.-S. Chen Y. Hutchins N. A. Ayala A. (2016). Contribution of Programmed Cell Death Receptor (PD)-1 to Kupffer Cell Dysfunction in Murine Polymicrobial Sepsis. Am. J. Physiol.-Gastrointest. Liver Physiol. 311 (2), G237–G245. 10.1152/ajpgi.00371.2015 27288425 PMC5007287

[B47] Wang M. Jo J. Song J. (2019). Adiponectin Improves Long-Term Potentiation in the 5XFAD Mouse Brain. Sci. Rep. 9 (1), 8918. 10.1038/s41598-019-45509-0 31222110 PMC6586823

[B48] Wu X. Lv Y.-G. Du Y.-F. Chen F. Reed M. N. Hu M. (2018). Neuroprotective Effects of INT-777 against Aβ1-42-Induced Cognitive Impairment, Neuroinflammation, Apoptosis, and Synaptic Dysfunction in Mice. Brain Behav. Immun. 73, 533–545. 10.1016/j.bbi.2018.06.018 29935310

[B49] Yao S. Wang S. Zhu Y. Luo L. Zhu G. Flies S. (2009). PD-1 on Dendritic Cells Impedes Innate Immunity against Bacterial Infection. Blood 113 (23), 5811–5818. 10.1182/blood-2009-02-203141 19339692 PMC2700320

[B50] Zhang X. Gou Y.-J. Zhang Y. Li J. Han K. Xu Y. (2020). Hepcidin Overexpression in Astrocytes Alters Brain Iron Metabolism and Protects against Amyloid-β Induced Brain Damage in Mice. Cell Death Discov. 6 (1), 113. 10.1038/s41420-020-00346-3 33298837 PMC7603348

[B51] Zheng J. Xie Y. Ren L. Qi L. Wu L. Pan X. (2021). GLP-1 Improves the Supportive Ability of Astrocytes to Neurons by Promoting Aerobic Glycolysis in Alzheimer's Disease. Mol. Metab. 47, 101180. 10.1016/j.molmet.2021.101180 33556642 PMC7905479

