# Supplementary material for: Programmed Cell Death Protein 1 Blockade Reduces Glycogen Synthase Kinase 3β Activity and Tau Hyperphosphorylation in Alzheimer’s Disease Mouse Models
Source: Front Cell Dev Biol. 2021 Dec 16;9:769229. doi: 10.3389/fcell.2021.769229 (PMC8716757; doi:10.3389/fcell.2021.769229)
Supplement: Supplementary file 1 [file DataSheet1.PDF]

Supplementary Information

Zou et al.

Programmed cell death protein 1 blockade reduces glycogen synthase kinase 3β activity and tau hyperphosphorylation in Alzheimer’s disease mouse models

Supplementary Table 1 Primary antibodies used in this study

Antibody	Dilution	Source	Identifier
**Mouse anti-PD1**	1:50 (Flow Cyt)	Biolegend	Cat#329908
**Rabbit anti-PD1**	1:300 (IHC); 1:1000(WB)	MilliporeSigma	Cat#PRS4065
**Mouse anti-PDL1**	1:50 (Flow Cyt)	Biolegend	Cat#393610
**Rabbit anti-PDL1**	1:200 (IHC); 1:1000(WB)	Abcam	Cat#ab213480
**Mouse anti-PDL1**	1:400 (IF)	Proteintech	66248-1-1g
**Rabbit anti-p-tau(Ser396)**	1:1000 (WB)	Anaspec	Cat#9455
**Mouse anti-p-tau (Thr231)**	1:1000 (WB)	Abcam	Cat#ab151559
**Mouse anti-tau**	1:1000 (WB)	Cell Signaling Technology	Cat#46687
**Mouse anti-GSK3β**	1:1000 (WB)	Abcam	Cat#ab32391
**Rabbit anti-GSK3β**	1:200 (IF)	Cell Signaling Technology	Cat#12456
**Rabbit anti-p-GSK3β (Ser9)**	1:1000 (WB/IHC)	Cell Signaling Technology	Cat#5558S
**Mouse anti-β-actin**	1:20000 (WB)	MilliporeSigma	Cat# A5441

WB, Western blotting; IHC, Immunohistochemistry; Flow Cyt, Flow Cytometry; IF, Immunofluorescence

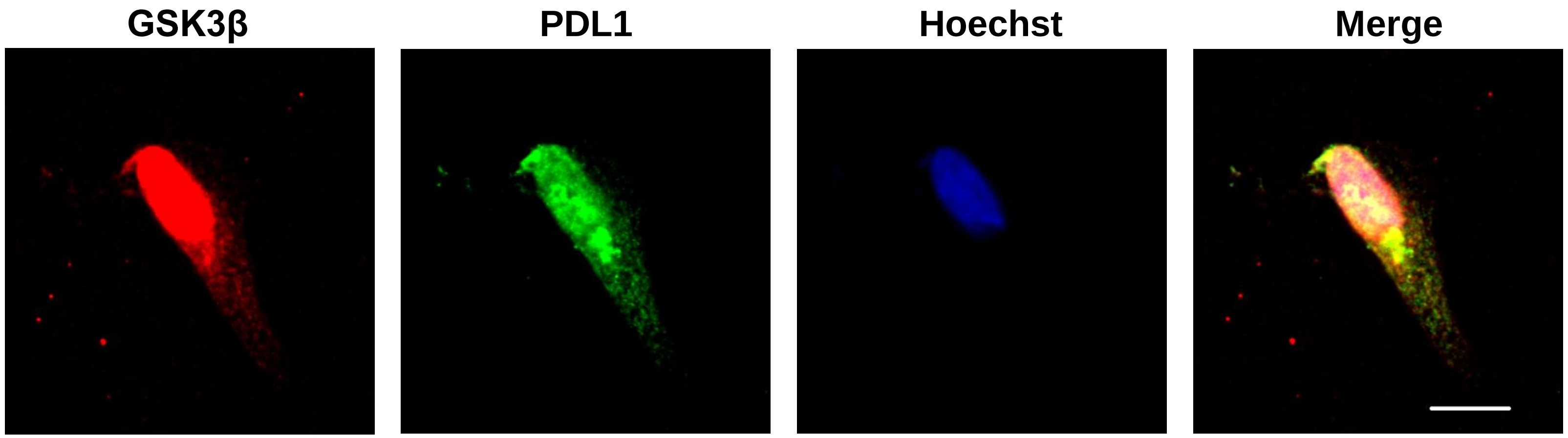

Supplementary Figure 1 Colocalization of PDL1 and GSK3β was observed in SH-SY5Y-APP cell. SH-SY5Y-APP cells were seeded on a coverslip precoated with 4% (w/v) poly-D-lysine for 24 h. Then, cells were fixed with 4% (v/v) paraformaldehyde for 20 min followed by 5% (v/v) triton-X 100 for permeabilization. 1% (w/v) BSA was added to cells to block nonspecific binding for 60 min after PBS washing. The cells were incubated with primary antibodies (PDL1, GSK3β) overnight at 4 ℃. After PBS washing, cells were incubated with Alexa Fluor 546 (anti-rabbit IgG), AlexaFluor 488 (anti-mouse IgG) conjugate secondary antibodies and hoechst (33342) at room temperature for 1 h. Each coverslip was then covered with about 20 ul mounting medium and analyzed on a laser scanning confocal microscope. The scale bars = 100 μm.
